# Developmental Changes of ENaC Expression and Function in the Inner Ear of Pendrin Knock-Out Mice as a Perspective on the Development of Endolymphatic Hydrops

**DOI:** 10.1371/journal.pone.0095730

**Published:** 2014-04-21

**Authors:** Bo Gyung Kim, Jin Young Kim, Hee Nam Kim, Jinwoong Bok, Wan Namkung, Jae Young Choi, Sung Huhn Kim

**Affiliations:** 1 Department of Otorhinolaryngology, Yonsei University, College of Medicine, Seoul, Korea; 2 Research Center for Natural Human Defense System, Yonsei University, College of Medicine, Seoul, Korea; 3 Division of Otology, Hana ENT Hospital, Seoul, Korea; 4 Department of Anatomy, Yonsei University, College of Medicine, Seoul, Korea; 5 College of Pharmacy, Yonsei institute of Pharmaceutical Sciences, Yonsei University, Incheon, Korea; Universidad Autonoma de San Luis Potosi, Mexico

## Abstract

Pendrin mutations cause enlarged vestibular aqueducts and various degrees of sensorineural hearing loss. The selective abolition of pendrin causes dilation of the membranous labyrinth known as endolymphatic hydrops, loss of the endocochlear potential, and consequently loss of hearing function. Because Na^+^ transport is one of the most important driving forces for fluid transport, the epithelial Na^+^ channel (ENaC) is believed to play an important role in fluid volume regulation in the inner ear. Therefore, the dysfunction of Na^+^ transport through ENaC by the acidification of endolymph in Pendred syndrome is one of the potential causes of endolymphatic hydrops. We investigated the changes of ENaC expression and function during the development of the pendrin knock-out mouse. In the cochlea, the expression of β and γENaC was significantly increased at P56 in *Pds^−/−^* mice compared with *Pds^+/+^* mice. In the vestibule, the expression of βENaC was significantly increased at P56, and γENaC expression significantly increased from P6 to P56 in *Pds^−/−^* mice. The ENaC-dependent trans-epithelial current was not significantly different between *Pds^+/+^* and *Pds^−/−^* mice in Reissner’s membrane or the saccular extramacular roof epithelium at P0, but the current was significantly increased in *Pds^−/−^* mice at P56 compared with *Pds^+/+^* mice. These findings indicate that the expression and function of ENaC were enhanced in *Pds^−/−^* mice after the development of endolymphatic hydrops as a compensatory mechanism. This result provides insight into the role of Na^+^ transport in the development and regulation of endolymphatic hydrops due to pendrin mutations.

## Introduction

**Figure 1:**
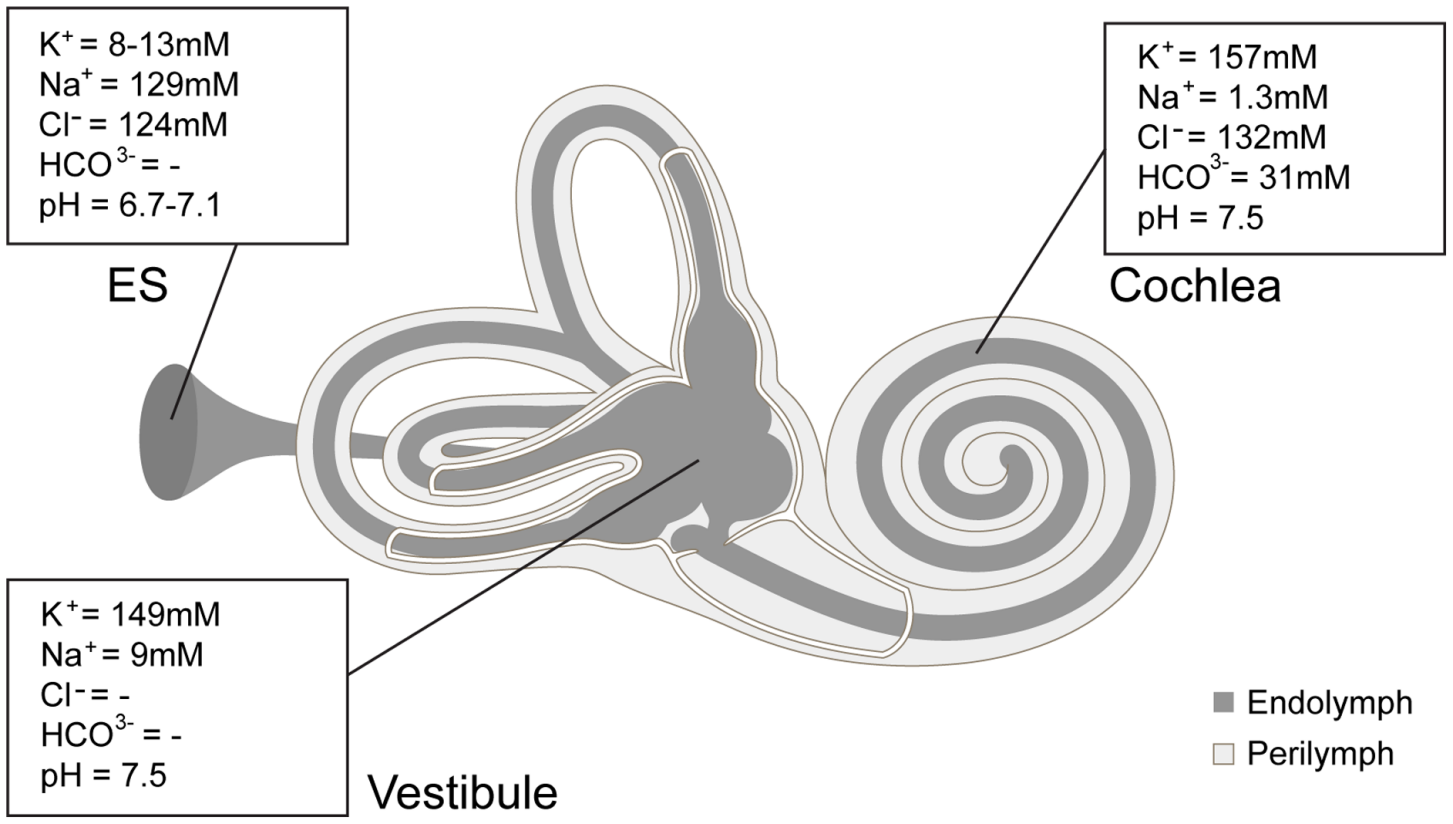
Schematic drawing of inner ear and fluid ion composition [33]. Dark gray area represents luminal space (endolymphatic space) filled with endolymph and light gray area represents abluminal space (perilymphatic space) filled with perilymph. ES; endolymphatic sac.

Pendrin is an anion exchanger that transports intracellular HCO_3_^-^, I^-^, and formate to the extracellular space in exchange for Cl^-^ [1]–[3]. The inner ear is composed of luminal space, which is surrounded by epithelial cells with tight junctions (membranous labyrinth), and abluminal space between the luminal space and surrounding bone (Fig. 1). The luminal space is called as the endolymphatic space and is filled with a fluid known as endolymph, which has a unique ion composition (high [K^+^], low [Na^+^], and nearly neutral pH) and is essential for maintaining normal hearing and balance. In the inner ear, pendrin is distributed to the following specific parts of the non-sensory epithelium of the membranous labyrinth: the spiral prominence, root cells, and spindle cells of the cochlea, vestibular transitional cells, and the endolymphatic sac epithelium [4], [5]. Pendrin plays an important role in endolymphatic pH regulation during embryonic development. The selective disruption of pendrin in mice causes the dilation of the membranous labyrinth (endolymphatic hydrops) beginning at 14.5 embryonic days, which can stretch the inner ear epithelial cells. Such stretching of the inner ear epithelium can cause the loss of endocochlear potential by the degeneration of the stria vascularis, which consequently causes the loss of hearing [6], [7]. In humans, pendrin mutation causes enlarged vestibular aqueducts, which is caused by the dilation of the membranous labyrinth, and various degrees in sensorineural hearing loss [8]. Thus far, the exact mechanism for the development of endolymphatic hydrops is not well understood, although it has been proposed that the acidification of endolymph causes a decrease in the H^+^-ATPase function in the endolymphatic sac, which then results in decreased Na^+^ absorption through ENaC and consequently causes endolymphatic hydrops [9].

The role of ENaC during development is likely important in the maintenance of a normal volume of endolymph. First, ENaC is distributed in extra-sensory epithelial cells, which compose an abundant surface area in the inner ear [10]; second, endolymph is composed of high [Na^+^] (∼140 mM) and low [K^+^] (∼1–4 mM) during embryonic development, which rapidly rises to high [K^+^] (∼140 mM) and low [Na^+^] (∼1–6 mM) over the first postnatal week [11]. If Na^+^ absorption through ENaC fails during the embryonic stage, endolymphatic hydrops can occur. Additionally, after the development of endolymphatic hydrops, the expression and function of ENaC can be changed in response to dilation of the endolymphatic space, which may be a compensatory mechanism.

In this study, we investigated the change of ENaC expression and function during the development of the pendrin knock-out mouse to elucidate the role of ENaC in the formation and regulation of endolymphatic hydrops.

## Methods

### Ethics Statement

All mice were treated in accordance with the guidelines for the Care and Use of Laboratory Animals of Yonsei University College of Medicine. All procedures involving animals were approved by the committee on Animal Research at Yonsei University College of Medicine (Approval reference number: #09-219).

### Tissue Preparation

*Pds^+/+^* and *Pds^−/−^* homozygous mice were obtained from colonies bred from heterozygous mice kindly provided by Eric D. Green [6]. *Pds^+/+^* and *Pds* ^−/−^ mice ranging from postnatal (P) day 0 to P56 (P0, P6, P15, P56) were used. The mice were anesthetized by i.p. injection of 30 mg/kg tiletamine-zolazepam (Zoletil, 500 mg/vial, Virbac, Carros, France) and 10 mg/kg xylazine (Rompun, Bayer, Leverkusen, Germany) and sacrificed by decapitation. Temporal bones were dissected, and the bony shells of the cochlea and vestibule were removed in Cl^–^free physiologic saline. The membranous portion of the cochlea and vestibule were separated, and each was used for real-time RT-PCR. For the ENaC-dependent trans-epithelial current measurement, we used two representative epithelial tissues of the cochlear and vestibular compartments: Reissner’s membrane and the non-sensory roof epithelium of the saccule, which were reported to show mostly ENaC-dependent trans-epithelial current at their apical side [10]. The membranous tissue of Reissner’s membrane and the extra-macular roof epithelium of the saccule from P0 and P56 mice were dissected into a perilymph-like physiologic saline solution and folded for measurement of the trans-epithelial current using a vibrating probe as reported previously [12], [13]. Whole vestibular tissue used in RT-PCR included more than the saccular extramacular epithelium, which was used for transepithelial current measurements. The results of real time RT-PCR were obtained from the semicircular canal duct epithelium, utricle, and saccule. Since the semicircular canal duct has been reported to exhibit ENaC on the apical side, ENaC expression in semicircular canal duct epithelium may have affected the RT-PCR results, and the transepithelial current from the saccular extramacular epithelium may not be completely representative of the rest of vestibule.

### RNA Isolation and Reverse Transcription

After homogenization of the cochlea and vestibule of *Pds^+/+^* and *Pds^−/−^* mice at each developmental period, total RNA was extracted using TRIzol (Invitrogen, Carlsbad, CA, USA) following the manufacturer’s protocol. The quantity and quality of isolated RNA were determined with a Nanodrop ND-100 spectrophotometer (Nanodrop Technologies, Wilmington, DE, USA) and by analyzing the 18 S and 28 S rRNA bands after electrophoresis, respectively. cDNA was synthesized from 3 µg of total RNA with random hexamer primers (Perkin Elmer Life Sciences, Boston, MA, USA and Roche Applied Science, Mannheim, Germany), AMV reverse transcriptase (Perkin Elmer Life Sciences), and RNase inhibitor (Perkin Elmer Life Sciences). The reverse transcription step was performed for 10 min at room temperature, 30 min at 50°C, and 15 min at 95°C.

### Real-time RT-PCR for α, β, and γ-ENaC

Real-time RT-PCR was used to compare the transcript levels of α, β, and γ-ENaC in the cochlea and vestibule between *Pds^+/+^* and *Pds^−/−^* mice. The transcripts of each ENaC subunit were amplified using gene-specific primers used in a previous study; the authors verified the identity of PCR products by sequencing purified PCR products (Table 1) [14].

**Table 1:** Primers used in real-time PCR [14].

Gene	GenBank Accession No.	Forward Primer	Reverse Primer	Amplicon Size (bp)
*8S rRNA*	BK000964	GAGGTTCGAAGACGATCAGA	TCGCTCCACCAACTAAGAAC	315
*Scnn1a*	NM_011324	AACGACCAAACGAACCFAACAC	GCTCAGAAGGCACACAAGAAGG	315
*Scnn1b*	NM_011325	CTCGGTGCTGTGCCTCATTG	GCCTCAGGGAGTCATAGTTGGG	278
*Scnn1g*	NM_011326	TGGTCCTCCTATCCTCGTTCTG	GTCACACCCATCAGGCAATAGC	344

*18S rRNA*, 18S rRNA; *Scnn1a, b,* and *g*, Na^+^ channel, nonvoltage-gated, type I, α, β, and γ (epithelial Na^+^ channel α, β, and γ).

Real-time PCR was performed on a 7300 Real-Time PCR System (Applied Biosystems, Foster City, CA, USA) with the DyNAmoHS SYBR Green qPCR kit (Finnzymes, Espoo, Finland) using 10 µl of cDNA in each well. The thermocycler parameters were 50°C for 2 min, 95°C for 10 s, and 40 cycles of 95°C for 15 s and 60°C for 1 min. To exclude the possibility of nucleotide contamination during PCR, no-template controls were performed and accepted when the C_t_ value was at least nine cycles greater than the template run. Measurements were performed in duplicate and accepted if the difference of C_t_ value between the duplicates was <1. The generation of a single product of appropriate size was checked by the presence of a single melt peak and by agarose gel electrophoresis. The specific gene expression was normalized to the level of 18S rRNA in each sample as described previously, with the fidelity of each PCR being taken into account [14].

### Trans-epithelial Current Measurement

The vibrating-probe technique was used to measure the trans-epithelial current of Reissner’s membrane and saccular extra-macular roof epithelium as previously described [12], [15]. Briefly, current density was monitored by vibrating a platinum-iridium wire microelectrode insulated with parlene-C (Micro Electrodes, Gaithersburg, MD, USA) and coated with Pt black on the exposed tip. The electrode tip of the probe was vibrated at two frequencies between 400 and 700 Hz along a horizontal (*x*) and vertical (*z*) axis by piezo-electric bimorph elements (Applicable Electronics, Forestdale, MA, USA) and was positioned 4±2 µm from the apical surface of the tissue membrane. The *x*-axis was perpendicular to the face of the epithelium. A platinum-black electrode served as reference in the bath chamber. The signals from the oscillators driving the probe, which were connected to a dual-channel phase-sensitive detector (Applicable Electronics), were digitized (16 bit) at a rate of 0.5 Hz. The electrode was positioned where current density showed a maximum *x* value and minimum *z* value; positive current values were defined as cation absorption/anion secretion and negative values were defined as cation secretion/anion absorption. The data derived from the x direction current density were plotted with Origin software, version 8.0. (OriginLab Software, Northampton, MA, USA).

For electrophysiological experiments, a perilymph-like physiological saline solution [150 mM NaCl, 3.6 mM KCl, 1 mM MgCl_2_, 0.7 mM CaCl_2_, 5 mM glucose, and 10 mM HEPES (pH 7.4)] was used for perfusion. Amiloride was purchased from Sigma (St. Louis, MO, USA) and dissolved in physiological saline solution before application.

### Statistical Analysis

Results are presented as the means ± SE from *n* observations. The differences of mRNA expression levels between *Pds^+/+^* and *Pds^−/−^* mice were evaluated by Mann-Whitney Rank Sum test. The current from the inner ear tissue of *Pds^+/+^* and *Pds^−/−^* mice was obtained from 30 to 45 seconds when the probe was located at the apical side of the membrane and during the last 30 seconds after 10 µM amiloride application, and the mean value of each current was calculated. Then, the mean values of trans-epithelial current between *Pds^+/+^* and *Pds^−/−^* mice were compared. The significance of the current density differences between *Pds^+/+^* and *Pds^−/−^* mice was calculated by Mann-Whitney Rank Sum test. The current density difference among P0, P6, and P56 groups for each genotype were evaluated by One Way ANOVA with Holm-Sidak or Dunn’s post-test. Each post test was clarified in the result. A value of *p*<0.05 was considered significant.

## Results

### Developmental Changes of ENaC Expression

**Figure 2:**
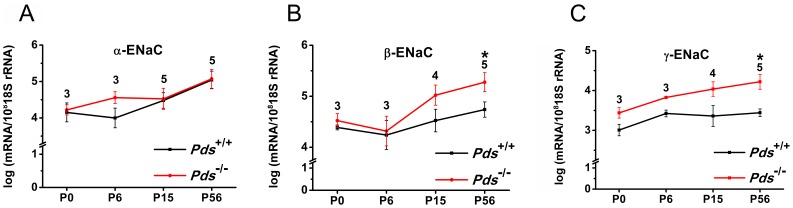
Differences of transcript expression of αENaC (A), βENaC (B), and γENaC (C) in the cochlea between *Pds^+/+^* and *Pds^−/−^* mice. The transcripts of β and γENaC were significantly upregulated at P56 in *Pds^−/−^* compared with *Pds^+/+^* mice. *p<0.05, numbers above the bars represent the number of experiments.

In both the cochlea and vestibule, the expression of β and γENaC increased more significantly during development in the *Pds^−/−^* compared with the *Pds^+/+^* mice, whereas the expression of αENaC was not significantly different between the groups during development. In the cochlea, the transcript expression of β and γENaC was significantly increased at P56 by 3.1- and 6.2-fold, respectively, in the *Pds^−/−^* mice (n = 5 for each β and γENaC, *p* = 0.045 and 0.008, respectively, Fig. 2).

**Figure 3:**
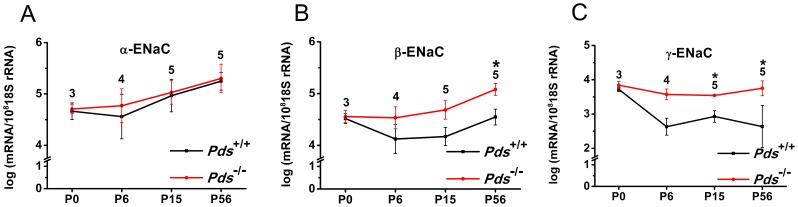
Differences of transcript expression of αENaC (A), βENaC (B), and γENaC (C) in the vestibule between *Pds^+/+^* and *Pds^−/−^* mice. The transcripts of βENaC and γENaC were significantly upregulated at P56 and from P6 to P56, respectively, in *Pds^−/−^* compared with *Pds^+/+^* mice. *p<0.05, numbers above the bars represent the number of experiments.

In the vestibule, the expression of βENaC was significantly increased at P56 by 3.0-fold in the *Pds^−/−^* mice (n = 5, *p* = 0.008). The expression of γENaC in the vestibule was significantly increased from P15 to P56 in *Pds^−/−^* mice [3.2-fold at P15 (n = 5, *p* = 0.008), 4.4-fold at P56 (n = 5, *p* = 0.029) for γENaC] (Fig. 3). No significant difference was observed in the expression of β or γENaC at P0 between the *Pds^−/−^* and *Pds^+/+^* mice (n = 3).

### Developmental Changes of Trans-epithelial Current in Inner Ear Tissue

**Figure 4:**
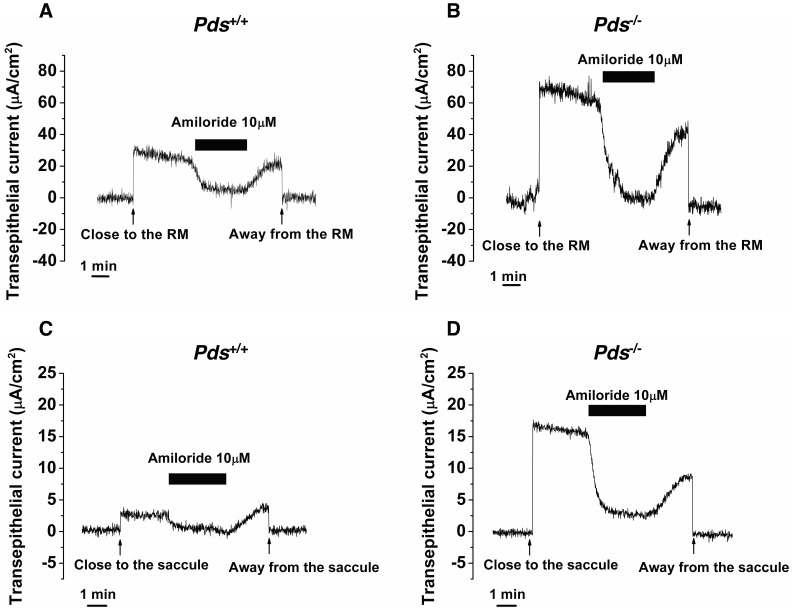
Representative figure of the difference of ENaC-dependent trans-epithelial current in Reissner’s membrane (A, B) and the saccular extramacular roof epithelium (C, D) between *Pds^+/+^* and *Pds^−/−^* mice at P56. RM; Reissner’s membrane.

We used P0, P6, and P56 mice to examine the functional difference of ENaC between *Pds^−/−^* and *Pds^+/+^* mice by measuring the ENaC-dependent trans-epithelial current. The ENaC-dependent current, which was mostly blocked by 10 µM amiloride, was detected in Reissner’s membrane and in the saccular extramacular roof epithelium of the *Pds^+/+^* and *Pds^−/−^* mice at P56 (Fig. 4).

**Figure 5:**
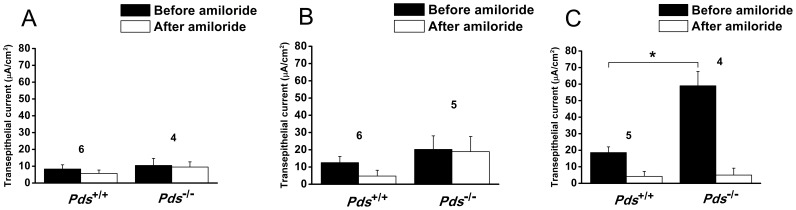
Differences of trans-epithelial current in Reissner’s membrane at P0 (A), P6 (B), and P56 (C). Cation absorption or anion secretion current was detected in *Pds^+/+^* and *Pds^−/−^* mice at P0 and P6 and was not significantly inhibited by 10 µM amiloride. The current was not significantly different between *Pds^+/+^* and *Pds^−/−^* mice. However, amiloride-sensitive current was detected at P56, and the current was significantly increased at P56 in *Pds^−/−^* compared with *Pds^+/+^* mice. *p<0.05, numbers above the bars represent the number of trans-epithelial current measurements.

In Reissner’s membrane, minimal cation absorption or anion secretion current was detected at P0 and P6, but the current was not inhibited by 10 µM amiloride [from 8.3±2.5 (before amiloride) to 5.7±2.1 µA/cm^2^ (after amiloride) in *Pds^+/+^* mice (n = 6, *p*>0.05) and 10.5±4.2 (before amiloride) to 9.5±3.1 µA/cm^2^ (after amiloride) in *Pds^−/−^* mice (n = 4, *p>0.05*) at P0 and from 12.6±3.6 (before amiloride) to 4.8±3.4 µA/cm^2^ (after amiloride) in *Pds^+/+^* mice (n = 6, *p*>0.05) and 20.2±7.9 (before amiloride) to 18.9±8.8 µA/cm^2^ (after amiloride) in *Pds^−/−^* mice (n = 5, *p*>0.05) at P6; *p*>0.05 for difference of basal current before amiloride application between *Pds^+/+^* and *Pds^−/−^* mice at P0 and P6 (Fig. 5)]. The trans-epithelial current at those periods was not significantly different between the *Pds^+/+^* and *Pds^−/−^* mice (*p*>0.05). However, trans-epithelial current was significantly increased at P56, and the current was nearly completely inhibited by 10 µM amiloride. In addition, the trans-epithelial current was significantly increased in the *Pds^−/−^* compared with *Pds^+/+^* mice [from 18.6±3.5 (before amiloride) to 4.2±3.0 µA/cm^2^ (after amiloride) in *Pds^+/+^* mice (n = 5, *p* = 0.03) and 59.0±8.7 (before amiloride) to 4.9±4.2 µA/cm^2^ (after amiloride) in *Pds^−/−^* mice (n = 4, *p* = 0.03), *p* = 0.02 for difference of basal current before amiloride application between *Pds^+/+^* and *Pds^−/−^* mice at P56 (Fig. 5)].

**Figure 6:**
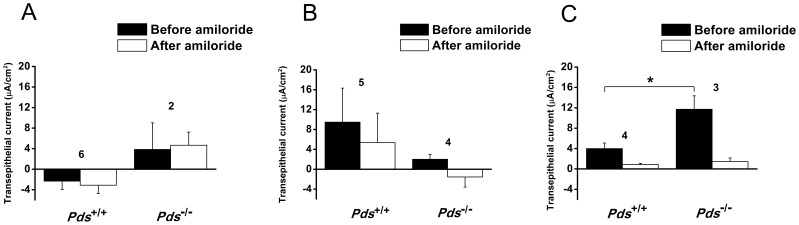
Difference of trans-epithelial current in the saccular extramacular roof epithelium at P0 (A), P6 (B), and P56 (C). The trans-epithelial current at P0 and P6 in the mice was not significantly inhibited by 10 µM amiloride. The current was not significantly different between the *Pds* ^+/+^ and *Pds* ^−/−^ mice. However, amiloride-sensitive current was detected at P56, and the current was significantly increased at P56 in the *Pds^−/−^* compared with *Pds^+/+^* mice. *p<0.05, numbers above the bars represent the number of trans-epithelial current measurements.

The trans-epithelial current of the extramacular roof epithelium of the saccule showed a similar tendency to that of Reissner’s membrane. The trans-epithelial current at P0 was very small, was not inhibited by 10 µM amiloride, and was not significantly different between the *Pds^+/+^* and *Pds^−/−^* mice [from −2.3±1.6 (before amiloride) to −3.1±4.7 µA/cm^2^ (after amiloride) in *Pds^+/+^* mice (n = 6, *p*>0.05) and 3.8±5.2 (before amiloride) to 4.7±2.6 µA/cm^2^ (after amiloride) in *Pds^−/−^* mice (n = 2, *p*>0.05), *p*>0.05 for difference of basal current before amiloride application between *Pds^+/+^* and *Pds^−/−^* mice at P0 (Fig. 6)]. At P6, the trans-epithelial current of the *Pds^+/+^* mice was increased and showed a cation absorptive or anion secretive current vector, which was not significantly inhibited by 10 µM amiloride [from 9.5±6.7 (before amiloride) to 5.3±5.9 µA/cm^2^ (after amiloride) in *Pds^+/+^* mice (n = 5, *p*>0.05) and 2.0±1.0 (before amiloride) to −1.5±2.0 µA/cm^2^ (after amiloride) in *Pds^−/−^* mice (n = 4, *p*>0.05), *p*>0.05 for difference of basal current before amiloride application between *Pds^+/+^* and *Pds^−/−^* mice at P6 (Fig. 6)]. The trans-epithelial current of the *Pds^−/−^* mice at P6 was still very small and was not different between P0 and P6 (Fig. 6). However, the trans-epithelial current was significantly increased in the *Pds^−/−^* mice at P56 compared with the *Pds^+/+^* mice and the current of both the *Pds^+/+^* and *Pds^−/−^* mice was mostly inhibited by 10 µM amiloride [from 4.0±1.1 (before amiloride) to 0.8±0.2 µA/cm^2^ (after amiloride) in *Pds^+/+^* mice (n = 4, *p* = 0.03) and 11.7±2.6 (before amiloride) to 1.5±0.7 µA/cm^2^ (after amiloride) in *Pds^−/−^* mice (n = 3, *p* = 0.02), *p = *0.02 for difference of basal current before amiloride application between *Pds^+/+^* and *Pds^−/−^* mice at P56 (Fig. 6)].

**Figure 7:**
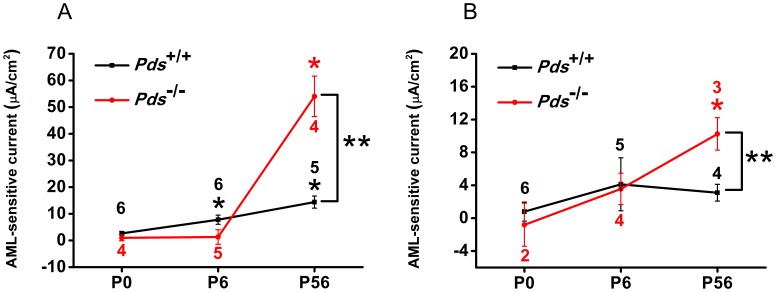
Difference of ENaC-dependent trans-epithelial current in Reissner’s membrane (A) and the extramacular roof epithelium of the saccule (B) at P0, P6, and P56. The ENaC-dependent current in Reissner’s membrane of *Pds^+/+^* and *Pds^−/−^* mice was significantly increased according to the development The current was not changed in saccular extramacular membrane of *Pds^+/+^* mice according to the development, but it was significantly increased at P56 of *Pds^−/−^* mice. The current difference between *Pds^+/+^* and *Pds^−/−^* mice was significant at P56 both in Reissner’s membreane and saccular extramacular membrane. [*p*<0.05 (One Way ANOVA with Holm-Sidak post-test) at P6 and P56 of *Pds^+/+^ mice* and *p*<0.05 (One Way ANOVA with Dunn’s post-test) at P56 of *Pds^−/−^* mice]. *p<0.05 in the current difference among P0, P6, and P56 groups for each genotype (One Way ANOVA with Holm-Sidak/Dunn’s post-test), **P<0.05 in the current density differences between *Pds^+/+^* and *Pds^−/−^* mice at P0, P6, and P56 (Mann-Whitney Rank Sum test), numbers above the bars represent the number of trans-epithelial current measurements.

We analyzed the pure ENaC-dependent current between the *Pds^+/+^* and *Pds^−/−^* mice by subtracting the current inhibited by amiloride from the current before amiloride application. The current was 2.6±0.7 (n = 6), 7.8±1.7 (n = 6), and 14.4±2.3 µA/cm^2^ (n = 5) at P0, P6, and P56, respectively, for Reissner’s membrane in *Pds^+/+^* mice and 0.9±1.1 (n = 4), 1.3±2.8 (n = 5), and 54.0±7.6 µA/cm^2^ (n = 4) at P0, P6, and P56, respectively, for Reissner’s membrane in *Pds^−/−^* mice; 0.8±1.2 (n = 6), 4.1±3.2 (n = 5), and 3.1±1.0 µA/cm^2^ (n = 4) at P0, P6, and P56, respectively, for the extramacular roof epithelium in *Pds^+/+^* mice and −0.8±2.6 (n = 2), 3.5±1.9 (n = 4), and 10.2±2.0 µA/cm^2^ (n = 3) at P0, P6, and P56, respectively, for the extramacular roof epithelium in *Pds^−/−^* mice (Fig. 7)].

The ENaC-dependent current in Reissner’s membrane of *Pds^+/+^* and *Pds^−/−^* mice was significantly increased according to the development [*p*<0.05 (One Way ANOVA with Holm-Sidak post-test) at P6 and P56 of *Pds^+/+^ mice* and *p*<0.05 (One Way ANOVA with Dunn’s post-test) at P56 of *Pds^−/−^* mice]. The current was not changed in saccular extramacular membrane of *Pds^+/+^* mice (One Way ANOVA, *p*>0.05), but it was significantly increased at P56 of *Pds^−/−^* mice (One Way ANOVA with Holm-Sidak post-test, *p*<0.05). The current difference was not significant between the *Pds^+/+^* and *Pds^−/−^* mice at P0 and P6 in Reissner’s membrane and extramacular roof epithelium of saccule (Fig. 7). However, the current was significantly increased in the *Pds^−/−^* mice at P56 compared with *Pds^+/+^* mice (*p* = 0.0009 for Reissner’s membrane and *p* = 0.04 for the saccule, Fig. 7). These results show that ENaC-dependent current developed after P6, and the increase in Na^+^ absorption via ENaC was greater after P6 in the *Pds^−/−^* mice than the *Pds^+/+^* mice.

## Discussion

Endolymphatic volume expansion (endolymphatic hydrops) is one of the significant changes in *Pds^−/−^* mice and resembles the human phenotype associated with the enlargement of the vestibular aqueduct. It has been reported that the area of scala media was enlarged by approximately 10-fold and that the spaces filled with perilymph were reduced in *Pds^−/−^* mice [16]. Endolymphatic hydrops is thought to cause impaired cell-to-cell communication, which results in the retarded growth of the organ of Corti and may damage the stria vascularis and ultimately lead to the deterioration of hearing [8], [17]. Vestibular function was significantly deteriorated in *Pds^−/−^* mice as assessed by the rotating rod measurements [3]. Endolymphatic hydrops is known to cause various insults in the inner ear, especially in *Pds^−/−^* mice, as described above; however, the mechanism for the development of endolymphatic hydrops in *Pds^−/−^* mice remains vague.

The lumen of the endolymphatic sac begins to form at E10.5, and the cochlear lumen is formed at approximately E14.5 in mice [9]. During the embryonic period, the Na^+^ concentration is higher than the K^+^ concentration in endolymph, a state that is reversed postnatally [11]. ENaC is believed to play an important role in regulating the Na^+^ concentration during development by reducing the Na^+^ concentration in endolymph postnatally, which finally results in high K^+^ and low Na^+^ concentration in endolymph. During embryonic development of the inner ear, ENaC expression was more significant in the endolymphatic sac than in any other compartment of the inner ear but was reduced in P2–5 [18]. Instead, ENaC expression was increased in the cochlea and vestibule during this period. This result implies that the Na^+^ concentration is primarily regulated in the endolymphatic sac during the embryonic period and that the main site of regulation is moved to the cochlea and vestibule postnatally. This inference is consistent with findings in our study, in which transcript expression and ENaC-dependent trans-epithelial current in the cochlea and vestibule tended to increase from P6 both in the *Pds^+/+^* and *Pds^−/−^* mice.

We investigated ENaC expression and function during the development of pendrin knock-out mice and demonstrated the role of ENaC in the inner ear as a compensatory mechanism for endolymphatic hydrops in the postnatal period. In this study, the expression and function of both β and γENaC transcripts tended to be increased in the inner ear from P6 and were significantly increased at P56 in the *Pds^−/−^* compared with *Pds^+/+^* mice. The endolymphatic hydrops and stretching of non-sensory epithelial cells are known to begin at E14.5 [8]. This may imply that fluid accumulation occurs from E14.5 and that there is a failure in the regulation of the endolymphatic Na^+^ concentration in the endolymphatic sac during that period. It was reported that the expression levels of βENaC rose between P4 and P8 in the cochlea of *Pds^+/−^* mice and γENaC expression rose between E17.5 and P0 in the cochlea of *Pds^+/−^* mice, which is earlier than *Pds^−/−^* mice [11]. And the Na^+^ concentration in the cochlea was similar at E16.5 between *Pds^+/−^* and *Pds^−/−^* mice but was higher in the *Pds^−/−^* than *Pds^+/−^* mice at P0 [11]. This result suggests that a Na^+^ absorption failure in the endolymphatic space occurs that may be caused by the decreased activity of ENaC in the endolymphatic sac by the acidification of endolymph. The acidification of endolymph around E15 decreases luminal vH^+^-ATPase function, which in turn caused the hypofunction of ENaC in the endolymphatic sac and decreased the driving force of Na^+^ absorption [9]. As a result, water cannot be absorbed in accordance with Na^+^ absorption, and endolymphatic hydrops can develop. However, in the postnatal period, as the main regulatory sites for the Na^+^ concentration of endolymph move to the cochlea and vestibule, the expression and function of ENaC in the cochlea and vestibule may be different between *Pds^+/+^* and *Pds^−/−^* mice. Regarding the postnatal increase in the transcript expression and function of ENaC in the cochlea and vestibule of *Pds^−/−^* mice, the mechanism can be explained in several ways. *In situ* condition, first, ENaC function could be increased by epithelium stretching that results from endolymphatic hydrops. ENaC function was reported to be increased by membrane stretching; thus, stretching forces in the inner ear epithelial cells could cause an increase in the function of ENaC [19]–[21]. However, this increase is caused by acute gating of channel and changes in the level of transcript expression cannot be explained. But, there was a report of increase of ENaC transcript expression in human urinary bladder epithelium with outlet obstruction which could cause chronic stretching of epithelial cells [22]. Therefore, chronic stretching of certain epithelial cell types including Reissner’s membrane and saccular extramacular membrane as well as bladder epithelium can cause increased transcript expression of ENaC. Second, increased expression and function of ENaC could be a compensatory mechanism for increased Na^+^ concentration in endolymph. The Na^+^ concentration in endolymph could still be higher in the *Pds^−/−^* than *Pds^+/+^* mice in the postnatal period. The Na^+^ concentration at P0 has been reported to be higher in the *Pds^−/−^* mouse than *Pds^+/+^* mouse, and the K^+^ concentration was lower in the *Pds^−/−^* mouse than *Pds^+/+^* mouse until P5 [11]. Thus, we hypothesized that the Na^+^ concentration could still be higher in the *Pds^−/−^* mouse than *Pds^+/+^* mouse from P0 to P3, when the main regulatory site for Na^+^ concentration of the inner ear moved from the endolymphatic sac to the cochlea and vestibule, and the ENaC expression and function was likely to be increased to compensate for increased endolymphatic Na^+^ concentration. To maintain the low Na^+^ concentration of endolymph in the *Pds^−/−^* mouse postnatally, an increase in the ENaC expression and function should be sustained. However, the increase of ENaC function was unlikely to be sufficient to overcome endolymphatic hydrops in *Pds^−/−^* mouse because hydrops is sustained during the postnatal period. This hypothesis can be supported by the fact that, endocochlear potential in the *Pds^−/−^* mice was absent [17]. This potential provides part of the driving force for cation absorption through ion channels in the apical membranes. Therefore, Na^+^ absorption through ENaC should be not as efficient as it is in *Pds^+/+^* mice. Third, human ENaC function was reported to be increased at an acidic pH by modulating Na^+^ self inhibition [23], [24]. It was revealed that this did not happen in the rat ENaC, but the function of mouse ENaC in acidic condition could be different from rat ENaC. Additionally, it was reported that extracellular Zn^2+^ enhanced mouse ENaC function by similar mechanism [25]–[27]. Therefore, acidic pH could affect mouse ENaC function. In adult *Pds^−/−^* mice, endolymph is acidic and this could increase cochlear and vestibular ENaC function. Additionally, extracellular domain of β and γENaC was reported to be required for the ENaC regulation by acidic pH, which can have influence on the transcriptional activity of β and γENaC in the *Pds^−/−^* mice as the result in our experiment [24]. However, this phenomenon was reported to be an acute gating mechanism of ENaC in acidic condition and there is no report about genomic effect of acidic pH on ENaC. Therefore, acidic condition cannot directly explain the increase of transcript expression level in *Pds^−/−^* mice.

Our *in vitro* experiment using vibrating probe cannot totally reflect *in situ* condition, because the excised membranes used in the experiment were not stretched same as *in situ*, and pH of the perfusion solution was nearly neutral, not acidic as *in situ*, in the current measurement in the inner ear of *Pds^−/−^* mice. Therefore, the results of our *in vitro* experiment can be explained only by the hypothesis that ENaC expression and function was increased for the compensation of increased endolymphatic Na^+^ concentration in *Pds^−/−^* mice and/or increased number of ENaC after chronic stretching of inner ear membrane.

Our results have possible clinical implications. In humans with Pendred syndrome, unlike knock-out mouse models, residual hearing is usually maintained after birth, although the degree of the residual hearing is variable depending on genotype [28]; pendrin function is partially preserved in humans, although the degree of hearing loss will vary. The residual hearing fluctuates or is aggravated during growth and is very sensitive to acoustic or physical trauma. Such traumas are hypothesized to cause functional damage to the distended epithelium or develop sudden fluid increase [29]. If the fluid in the endolymphatic space can be reduced, it can alleviate the damage to the inner ear epithelium, and this can be achieved by enhancing ENaC function. Enhancing ENaC function in humans may be more effective in reducing endolymphatic hydrops than in the mouse models because endocochlear potential could be preserved to some extent and could effectively cause the efflux of Na^+^ from the endolymphatic space to the perilymphatic space.

Steroids have been used to treat hearing fluctuation in patients with pendrin mutations. Intervention with corticosteroid therapy is crucial for preventing the residual hearing from further deterioration [30]. Patients with enlarged vestibular aqueducts who develop hearing impairment have a high rate of hearing improvement when treated with corticosteroids [31]. Steroids are known to enhance ENaC expression and function in the inner ear tissues, such as the mouse Reissner’s membrane, the rat semicircular canal duct epithelium, and the mouse saccular extramacular non-sensory epithelium [13], [14], [32]. These findings combined with our results suggest the possibility of using steroids in the treatment of hearing aggravation in Pendred syndrome patients. Recently, the local application of steroids (intratympanic) for the treatment of inner ear disorders is increasing, and this treatment can minimize the systemic adverse effects and allow frequent use. However, further investigation is needed for the direct evidence of this hypothesis.

## Conclusions

Both the transcriptional level and function of ENaC in the cochlea and vestibule significantly increased in *Pds^−/−^* compared with *Pds* ^+/+^ mice at P56. It is tempting to speculate that increased Na^+^ concentration in endolymph caused endolymphatic hydrops in the *Pds^−/−^* mice, and ENaC expression and function likely increased postnatally to compensate for the increased Na^+^ concentration of endolymph in the cochlea and vestibule. Based upon these findings, the cochlea and vestibule may be the main regulatory site of Na^+^ concentration in the inner ear postnatally, and this result provides insight into the role of ENaC in regulating fluid volume and Na^+^ homeostasis during development, which suggests the possibility of preserving hearing in Pendred syndrome patients by modulating ENaC function.
